# An Atraumatic Femoral Fracture in a Patient with Rheumatoid Arthritis and Osteoporosis Treated with Denosumab

**DOI:** 10.1155/2013/249872

**Published:** 2013-12-07

**Authors:** J. Villiers, D. W. Clark, T. Jeswani, S. Webster, A. L. Hepburn

**Affiliations:** ^1^Department of Rheumatology, Worthing Hospital, Worthing, UK; ^2^Department of Orthopaedic Surgery, Worthing Hospital, Worthing, UK; ^3^Department of Radiology, Worthing Hospital, Worthing, UK

## Abstract

Osteoporosis is responsible for a significant burden both individually and socially, but is readily treated with antiresorptive agents and mineral supplementation. However, long-term usage of these agents, notably bisphosphonates, is rarely associated with atypical fractures. Denosumab is a monoclonal antibody that reduces osteoclast activity and thus increases bone mineral density. In this case report, we present a 78-year-old woman with a background of rheumatoid arthritis and osteoporosis who presented with an atypical diaphyseal femoral fracture.

## 1. Introduction

Osteoporosis is a significant cause of morbidity and mortality. Following a neck of femur fracture, there is up to 50% loss of independence [1] and 20% mortality in the first 12 months [2]. Pharmacological therapy has been shown to significantly reduce fracture risk and therefore the subsequent morbidity and mortality [3].

Denosumab is a fully humanized monoclonal immunoglobulin that binds the receptor activator of nuclear factor-*κ*B ligand (RANK-L), a member of the tumour necrosis factor cytokine family, essential for the formation, function, and survival of osteoclasts [4]. Denosumab is thought to sequester RANK-L, preventing it from activating NF-*κ*B and subsequently reducing the resorption of bone.

In the United Kingdom, Denosumab is recommended by the National Institute for Health and Care Excellence (NICE) for the treatment of postmenopausal women with osteoporosis who are unable to tolerate or comply with the administration of oral bisphosphonates, which remains the first line treatment option. However, since the introduction of antiresorptive therapy in the management of osteoporosis, concern has arisen that these medicines are associated with atypical fractures with long-term use [5].

The extension of the phase 3 FREEDOM trial, which had shown the benefits of Denosumab in the treatment of osteoporosis [6], has suggested that atypical femoral fractures do rarely occur after prolonged exposure. In this case report we present a 78-year-old female with a history of rheumatoid arthritis and osteoporosis who presented with an atraumatic diaphysial fracture of her right femur.

## 2. Case Report

A 78-year-old female had been followed up in our Rheumatology outpatient clinic since being diagnosed with rheumatoid arthritis in 1987. Treatment has included occasional intraarticular and intramuscular corticosteroid injections, but never long-term oral corticosteroids. Current immunosuppressive therapy consists of methotrexate and hydroxychloroquine. She also has a background history of an idiopathic kyphoscoliosis, bronchiectasis, and hypertension. She had undergone bilateral total knee arthroplasties in 2007. Osteoporosis was diagnosed in March 2010, when a DEXA scan (Hologic, Crawley, United Kingdom) showed *T* scores in the lumbar spine (total) and left hip (total) of −2.7 and −2.9, respectively. She has no history of a prior fragility fracture. She was initially treated with oral alendronate, but after a few weeks of intermittent use, this drug was discontinued due to upper gastrointestinal intolerance. She was subsequently treated with Strontium ranelate, but stopped this after a few days due to lower gastrointestinal intolerance. She has also had great difficulty tolerating oral calcium and vitamin D supplements. Other relevant drug therapies on admission included Omeprazole 20 mg once daily, which she had been taking for two years. She was commenced on Denosumab in September 2011. She received a total of 3 doses of Denosumab 60 mg, given subcutaneously at 6 monthly intervals, the last dose being given in September 2012.

**Figure 1:**
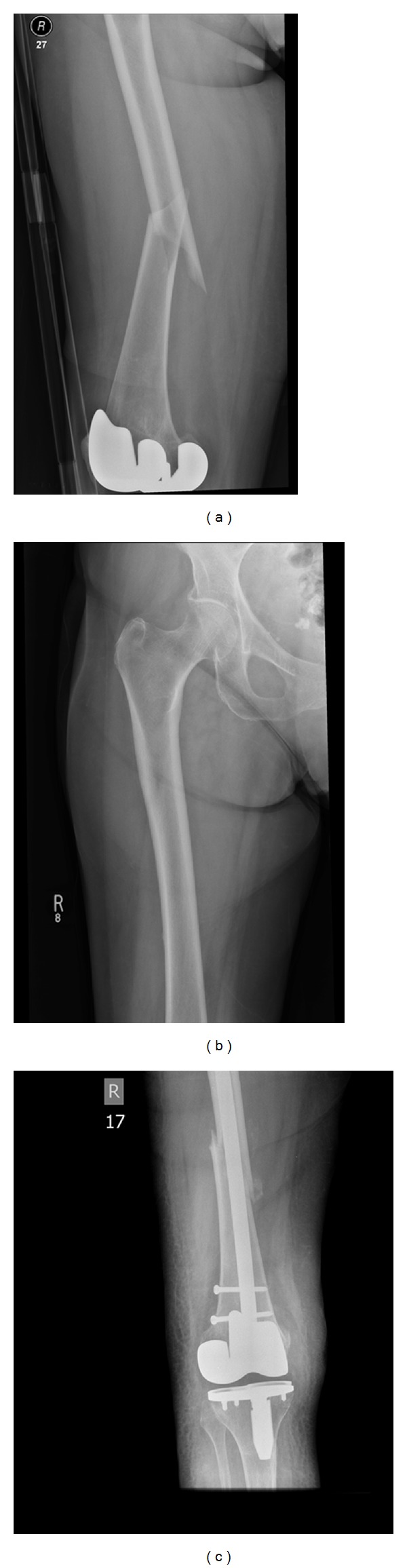
(a) Admission radiograph showing noncomminuted fracture with medial spike. (b) Radiograph taken 4 weeks prior to admission revealing subtle periosteal reaction on lateral cortex. (c) Radiograph 12 days postoperative repair.

She presented to our hospital in January 2013 reportedly attempting to stand from sitting on the side of her bed when she heard a crack and felt severe pain in her right thigh. After this, she became unable to bear weight as a result of severe pain. Importantly, she reported that for approximately five weeks prior to this, she had been experiencing increasing pain in both her thighs, more severe on the right, and that she had been increasingly dependent on crutches to mobilise. A radiograph of her right femur was requested on her admission to hospital, which revealed a noncomminuted spiral fracture with a medial spike (Figure 1(a)).

Four weeks prior to this admission, a radiograph of her right femur had been requested which was reported to have no abnormality seen with correct positioning of the knee prosthesis and no sign of loosening or a stress reaction (Figure 1(b)). However, a further review of this radiograph raised the possibility of a subtle periosteal reaction on the lateral cortex of the diaphysis. Blood tests on admission revealed that her albumin-adjusted calcium serum was 2.35 mmol/L (reference range 2.1–2.7 mmol/L), phosphate was 1.11 mmol/L (0.7–1.5 mmol/L), alkaline phosphatase was 68 *μ*mol/L (39–150 *μ*mol/L), and creatinine was 73 *μ*mol/L (60–120 *μ*mol/L). She reported no trauma immediately preceding or in the weeks before developing the fracture.

Management of her fracture was complicated as she was found to be suffering from type 2 respiratory failure that necessitated a short period in intensive care unit (ICU) where she was intubated and ventilated. On the following day, after she was stabilized, operative fixation of her femoral fracture was undertaken. An intramedullary nail was inserted (Figure 1(c)) and she was transferred back to ICU where she continued to wean off the bilevel positive airways pressure support and was treated with antibiotics.

Since her discharge, she has been reviewed in the Orthopaedic and Rheumatology outpatient clinics. The intramedullary nail maintains correct alignment, with moderate callus formation (Supplementary Figure 1(d) in Supplementary material available online at http://dx.doi.org/10.1155/2013/249872). There is cortical thickening in the contralateral femur (Supplementary Figure 1(e) in Supplementary material). The patient is now mobilizing with one stick and is taking a nonprescribed vitamin D supplement providing 800 iu daily. Further blood tests showed an albumin-adjusted serum calcium of 2.21 mmol/L, phosphate of 1.24 mmol/L, alkaline phosphatase of 100 *μ*mol/L, ESR of 48 mm/hr, normal protein electrophoresis, and a serum 25-OH vitamin D level of 65 nmol/L (70–200 nmol/L). No evidence of malignancy was observed on histological examination of bone samples taken at the time of surgery. A repeat DEXA scan in June 2013 shows a stable *T* score in the lumbar spine of −2.4 and a *T* score in the left hip of −2.9. Bone turnover markers were not done as they are not currently available in our laboratory. Treatment with Denosumab has been ceased indefinitely in accordance with current recommendations published by the Medicines and Healthcare products Regulatory Agency (MRHA) [7].

## 3. Discussion

Osteoporosis has a huge social and individual impact, which can be reduced by medical therapy. While evidence for bone protection in the short and medium term is strongly in favor, there are concerns that long-term usage is rarely associated with detrimental events.

The extension of the FREEDOM trial states the incidence of atypical femoral fractures as occurring 1–10 : 10000 patients treated with Denosumab 60 mg for greater than 2.5 years. The American Society for Bone and Mineral Research (ASBMR) task force was commissioned after concerns were raised with bisphosphonate usage and atypical fractures and they outlined several major and minor criteria for the diagnosis of atypical fractures [5].

The presented case is in agreement with the majority of the proposed criteria for an atypical femoral fracture [5]. Most importantly in this case, there was no history of trauma. Although spiral rather than transverse, it was noncomminuted and exhibited a medial spike (Figure 1(a)). We feel a subtle increase in cortical thickness is present in the diaphysis of both femurs (Supplementary Figures 1(b) and 1(e) in supplementary material) and delayed healing is apparent (Supplementary Figure 1(d) in supplementary material). Furthermore, prodromal symptoms were present in both thighs and the patient had a history of rheumatoid arthritis, treatment of which had included intermittent corticosteroids. This patient had received three doses of Denosumab, but had only received oral bisphosphonates for a very short period.

The underlying pathophysiology is not fully understood, but atypical fractures share clinical and radiological similarities with stress fractures [8]. Stress fractures typically occur following repeated trauma and are felt to be due to accumulation of microfractures. Radiological features of stress fractures include periosteal callus, which has been seen on the lateral cortex prior to atypical fractures occurring. In the presented case, a periosteal reaction was apparent 4 weeks prior to fracture on the lateral cortex of the right diaphysis (Figure 1(b)). Measurements confirm that the periosteal reaction is exactly where the lateral fracture line occurred.

At the molecular level, the organic matrix, largely consisting of collagen, is responsible for the vast majority of the bones strength and maturation of the collagen cross linkage which are felt to stabilize the matrix. It is this principle, which is utilized when prescribing an antiresorptive agent as they allow the collagen cross linkages time to mature. There is mounting evidence that the prolonged slowing of bone remodeling is allowing microfractures to accumulate and these, over time, can weaken bone eventually leading to macrofractures such as those presented in the case [5]. It is also interesting that this proposed mechanism also accounts for callus formation of the cortex prior to fracture as the bone attempts to heal the damage.

At the time of writing, this is only the second case of an atypical femoral fracture occurring in a patient with low bone density receiving Denosumab. Parapodis et al. [9] have recently reported a case of an 81-year-old woman with osteopenia on DEXA scanning on a background of chronic kidney disease and hyperparathyroidism. As in our case, there was no history of trauma but the described fracture was different in that it was subtrochanteric and transverse in configuration. Their patient had not received bisphosphonates, because of renal impairment, but had only received one dose of denosumab. This questions a direct causal relationship between the fracture and treatment with denosumab in this particular case.

The MRHA has recommended that all patients commenced on an antiresorptive agent should be informed of the risk of such fractures occurring and instructed to seek a medical assessment if they develop new onset thigh, hip, or groin pain [7]. They also recommend in the interim while they are being assessed that denosumab therapy should cease. It is also pertinent that both femurs are assessed as frequently atypical fractures occur bilaterally.

Further management of our patient's osteoporosis will be difficult. Use of a more potent parenteral bisphosphonate such as Zoledronate is a consideration, but concern remains given the association between atypical femoral fractures and treatment with bisphosphonates. Additionally, Paul et al. retrospectively looked at fracture healing in the context of bisphosphonates and found a 21% fixation failure rate compared to 3% for those not on bisphosphonates [10]. The use of Teriparatide to promote fracture healing has recently been described in patients with atypical femoral fractures [11]. Teriparatide alone is a treatment option, but its use is restricted in the UK by NICE and daily injection is necessary. This raises further concerns in a frail elderly patient with advance rheumatoid arthritis in terms of compliance with treatment given the dexterity required to self-administer the drug on a daily basis. We continue to encourage appropriate lifestyle measures to help improve bone density and prevent falls in this case, including compliance with a suitable preparation of oral calcium/vitamin D.

Atypical femoral fractures remain rare. Although an association with bisphosphonates is now recognized, the majority of cases still occur in patients not receiving drugs affecting bone metabolism. However, with increasing use of potent antiresorptive drugs, the number of reported cases is likely to increase. We advocate the advice given by the MHRA [7] and ASBMR [5] until further evidence becomes available regarding the long-term safety of these drugs.

## Supplementary Material

Figure 1d: Radiograph several weeks post operative repair showing callus formation.
Figure 1e: Radiograph of contralateral femur showing thickening of the cortex.
