# Supplementary material for: An Atraumatic Femoral Fracture in a Patient with Rheumatoid Arthritis and Osteoporosis Treated with Denosumab
Source: Case Rep Rheumatol. 2013 Dec 7;2013:249872. doi: 10.1155/2013/249872 (PMC3893843; doi:10.1155/2013/249872)
Supplement: Supplementary file 1 — Figure 1d: Radiograph several weeks post operative repair showing callus formation. Figure 1e: Radiograph of contralateral femur showing thickening of the cortex. [file 249872.f1.docx]

Supplimentary material.


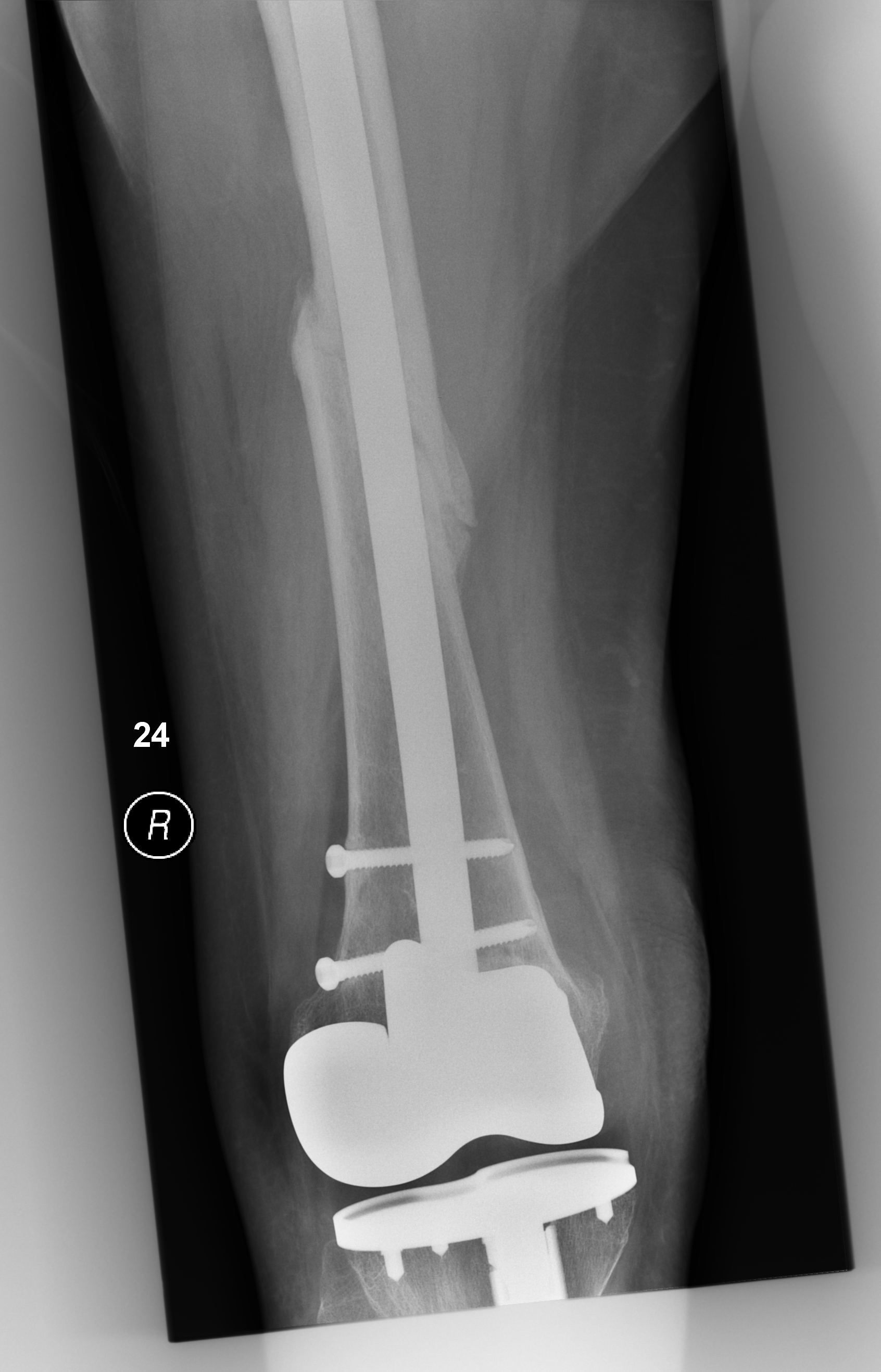


Figure 1d: Radiograph several weeks post operative repair showing callus formation.


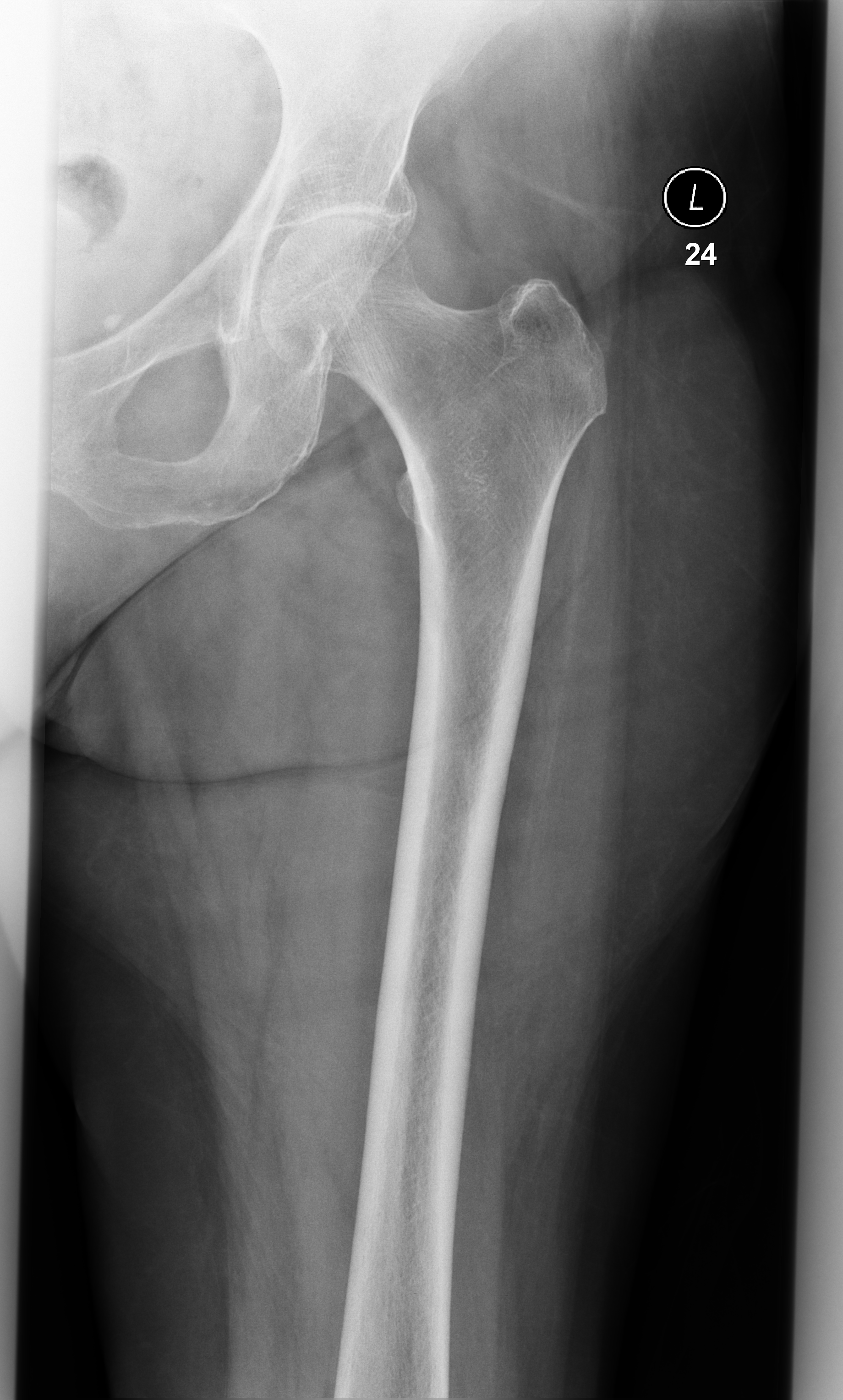


Figure 1e: Radiograph of contralateral femur showing thickening of the cortex.
